# A case of recurrent laryngeal nerve paralysis caused by radiofrequency ablation for mediastinal recurrence of lung cancer

**DOI:** 10.1016/j.radcr.2023.12.059

**Published:** 2024-01-16

**Authors:** Kazuki Murai, Akira Yamamoto, Ken Kageyama, Mariko Nakano, Atsushi Jogo, Yukio Miki

**Affiliations:** Department of Diagnostic and Interventional Radiology, Graduate School of Medicine, Osaka Metropolitan University, 1-4-3 Asahimachi, Abenoku, Osaka 545-8585, Japan

**Keywords:** Interventional radiology, Radiofrequency ablation, Lung cancer, Complication, Nerve injury, Recurrent laryngeal nerve

## Abstract

Radiofrequency ablation (RFA) has emerged as a potent therapeutic modality for tumor treatment, and offers benefits such as reduced recovery time and minimal damage to nearby tissues. However, RFA is not devoid of complications, notably nerve damage during intrathoracic lesion treatments, which can significantly impact patients' quality of life. This report describes the unique case of a 71-year-old male who experienced hoarseness attributed to injury to the recurrent nerve after RFA for a locally recurrent lung cancer lesion in the mediastinum near the aortic arch. Although RFA has the advantages of a minimally invasive nature and positive outcomes, its risk of nerve injury, specifically in the thoracic region, highlights the need for improved techniques and preventive measures.

## Introduction

Radiofrequency ablation (RFA) is widely recognized as an effective therapy for the treatment of tumors in various organs [1,2]. RFA has been reported to have certain favorable therapeutic effects, even in the treatment of primary lung cancer [3]. The advantages of this therapy include a shorter recovery period for the patient compared to surgery, elimination of the risk of general anesthesia, and minimal damage to the surrounding normal tissues [1,4].

However, RFA of the thoracic region carries the risk of complications specific to percutaneous treatment, including pneumothorax, hemothorax, infection and pleurisy [5]. There is particular concern regarding the risk of damage to nearby nerve tissue during RFA treatment of intrathoracic lesions [6]. Such nerve damage can cause permanent pain and functional disability after treatment. In addition, further therapeutic intervention and long-term rehabilitation may be required, which can significantly impact the patient's quality of life.

Nerve injuries reported after RFA in the thoracic region include intercostal nerve injury [6], brachial nerve injury [7], and phrenic nerve injury [8], with only a few reports of recurrent laryngeal nerve injury. We report a case of hoarseness that was caused by RFA treatment of a locally recurrent lung cancer lesion in the aortic arch area.

## Case report

**Fig. 1:**
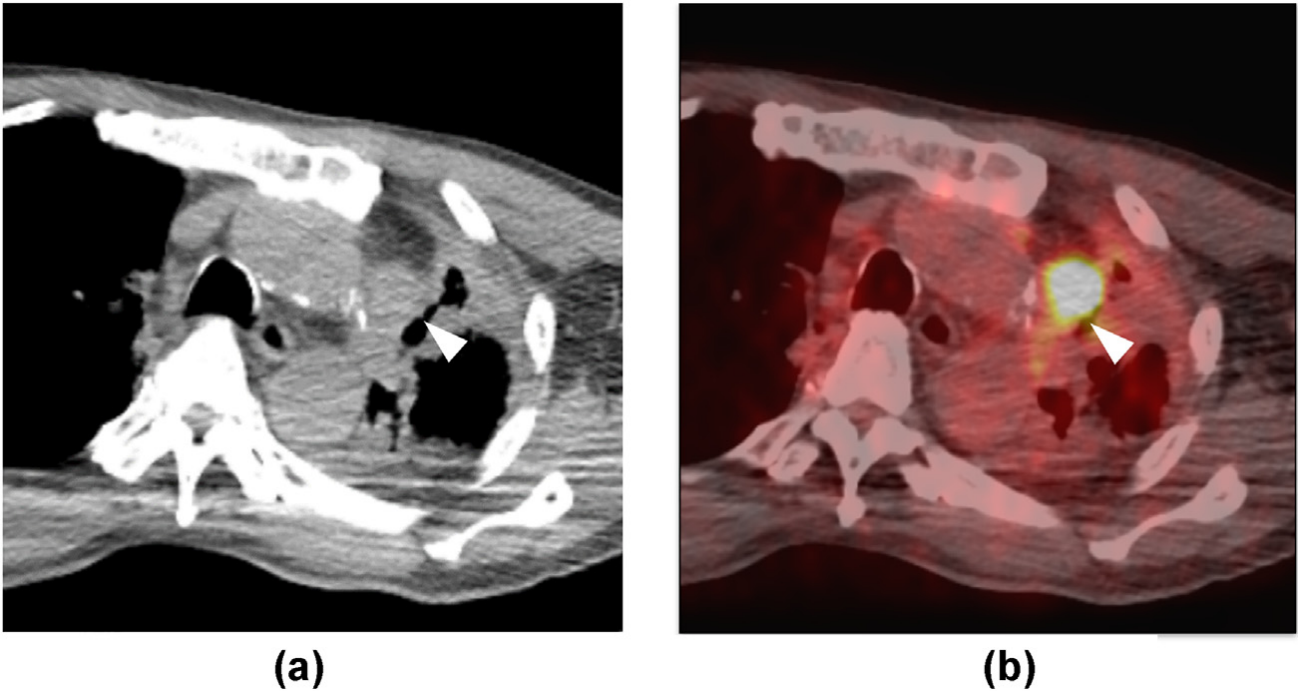
Pretreatment CT image. A nodule 20 mm in diameter (arrowhead) is seen on the mediastinal aspect of the left lung. The lesion abuts the aorta (A). FDG-PET/CT shows radionuclide uptake in the same lesion (B, arrowhead).

**Fig. 2:**
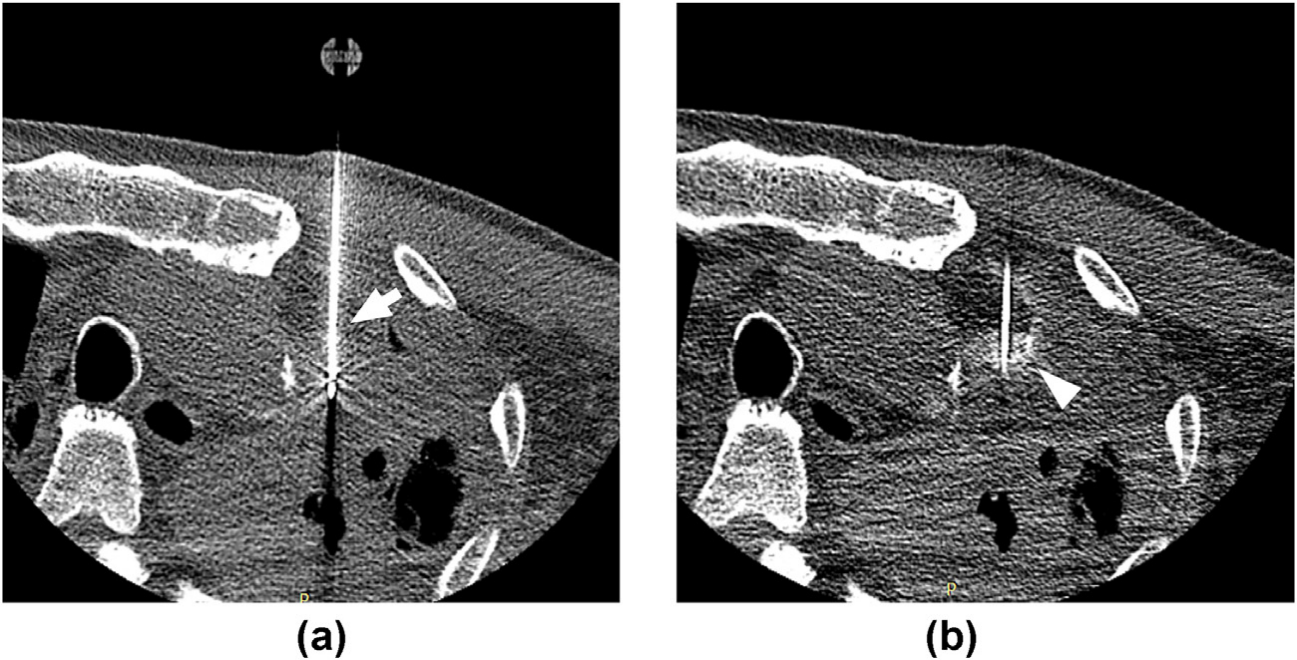
CT images during the RFA procedure. The tumor is punctured from the anterior chest wall with a Cool-Tip 2-cm electrode (A, arrow). Injected xylocaine mixed with contrast medium is seen as high density area around the tumor. The xylocaine mixture remains in the anterior aspect of the mass (arrowhead), which could not be separated from the aorta (B).

A 71-year-old male was diagnosed with lung cancer of the left upper lobe of the lung and underwent surgery following chemoradiotherapy (CRT). Five years after surgery, a locally recurrent lesion abutting the aortic arch was noted. Despite administration of second concurrent CRT, the lesion remained. After 5 years of chemotherapy, no new metastases were detected, but the locally recurrent lesion remained (Fig. 1). At 10 years after surgery (5 years after the second CRT), RFA was planned because further radiation therapy and surgical salvage therapy were considered difficult after 2 rounds of radiation therapy. RFA was performed under CT guidance, using a Cool-Tip 2-cm electrode (Covidien Inc, Boulder, CO). For pain relief and to separate the tumor from surrounding organs, an 18G needle was inserted between the tumor and pleural surface and 1% xylocaine mixed with a small amount of contrast agent was injected (Fig. 2). Although the separation was difficult due to adhesion between the tumor and pleura, the tumor was finally punctured through the anterior chest wall, and then was ablated until the break (automatic termination of the generator due to increased impedance) was achieved.

**Fig. 3:**
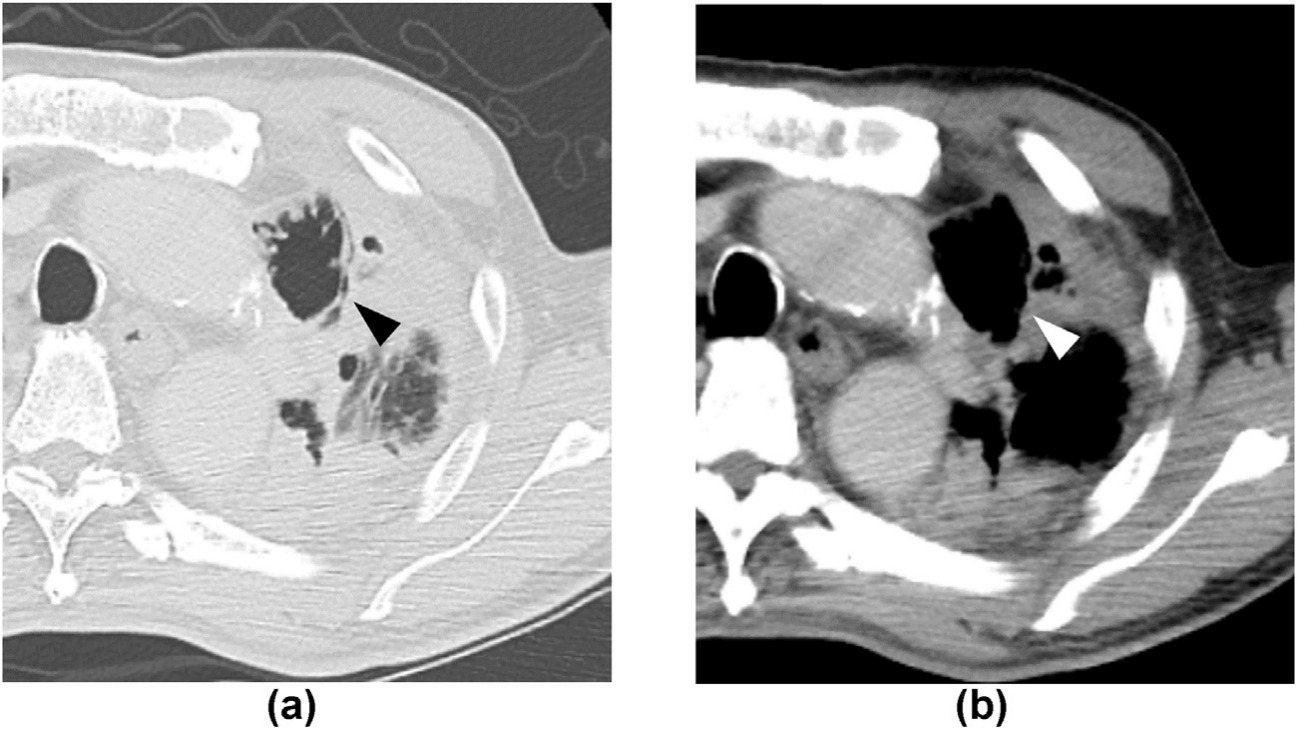
CT image after 6 months of treatment. The lesion has shrunk relative to the size on the pretreatment images. Cavitation is apparent with no signs of infection (A, B, arrowheads).

The patient developed hoarseness of breath at 1 month after treatment. Laryngoscopy revealed fixation of the left vocal cord, consistent with a diagnosis of left recurrent laryngeal nerve palsy. During 7 months of follow-up, there was only a slight improvement in symptoms and the hoarseness remained. The locally recurrent lesion reduced in size and them, formed a cavity (Fig. 3). There were no signs of infection and the lesion did not regrow during the 7 months of observation period.

## Discussion

Malignant diseases in the cervicothoracic region and aortic aneurysms are the main causes of recurrent laryngeal nerve paralysis [9], which has also been reported as a complication after surgery in the thoracic region [10]. Direct invasion in these situations cause damage to the recurrent laryngeal nerve. However, there are limited reports regarding injury of the recurrent laryngeal nerve by RFA, a percutaneous treatment. Although RFA has received attention as a minimally invasive alternative therapy for tumor treatment, and many favorable outcomes have been reported [1], [2], [3], [4], this therapy also can result in nerve injury. In this report, we describe a case of recurrent laryngeal nerve injury following RFA treatment for local recurrence of a lung cancer in the aortic arch area.

Complications specific to percutaneous treatment have been reported for RFA, among which is nerve injury [5]. Intercostal, brachial, and phrenic nerve injuries have occurred after RFA in the thoracic region [6], [7], [8]. However, there are few reports of recurrent laryngeal nerve injury from RFA limited to the thoracic region. A retrospective review of nerve injury during lung RFA reported only 1 case of recurrent laryngeal nerve injury [6], and the incidence of this complication is unknown. In the only previous report of recurrent laryngeal nerve injury, RFA of a lesion at the pulmonary apex was thought to have injured the recurrent laryngeal nerve near the superior mediastinum [6]. In our case, the nerve injury was caused by RFA of a lesion abutting the aortic arch.

Anatomically, the recurrent laryngeal nerve has a unique and somewhat circuitous path. The recurrent laryngeal nerve descends from the neck to the superior mediastinum, then winds around the subclavian artery on the right side and the aortic arch on the left side before ascending again [11]. Because of this unique pathway of the recurrent laryngeal nerve, even therapeutic interventions in the thoracic region sometimes result in unexpected laryngeal neuropathy. As in our case, it should be noted that ablation around the aortic arch also potentially risks paralysis of the recurrent laryngeal nerve.

In RFA, tissue is heated to a high temperature, which can also affect the surrounding nerve tissue. Past experimental reports have shown that transient damage occurs at 47°C and irreversible damage occurs at 50°C and higher [12,13]. Nerve damage is thought to occur in a temperature-dependent manner. In thermal coagulation with RFA, the temperature commonly exceeds 80°C. Therefore, nerve damage after RFA is often irreversible, and recovery may be difficult once such damage occurs. In the present case, the recurrent laryngeal nerve was presumably damaged during thermal coagulation of the tumor, resulting in persistent hoarseness.

There is currently no literature on the treatment of recurrent laryngeal nerve palsy with RFA in the thoracic region. Surgical traction or electrocautery can sometimes cause postoperative recurrent laryngeal nerve palsy. In such cases, laryngoplasty or arytenoid adduction is considered only when symptoms significantly impact the patient's daily life. Generally, many patients experience symptom improvement within several months due to the compensatory effect of the contralateral vocal cord, and speech therapy and rehabilitation are effective. Rehabilitation is especially important for the elderly because of the risk of aspiration due to laryngeal dysfunction [10,14]. As observed in our case, nerve damage from the prolonged high temperatures by RFA can lead to permanent symptoms, necessitating careful consideration.

Accordingly, procedural planning, such as confirmation of lesion anatomy and use of technical preventives, is important to minimize the risk of nerve damage when performing RFA in the thoracic region. Hydrodissection and artificial pneumothorax are well-known methods [15]. In our case, hydrodissection was performed with the aim of separating the lesion from the surrounding tissue, but separation from the aortic arch was difficult. Although the RFA treatment resulted in hoarseness, if no additional treatment had been administered and the tumor grew, it is likely that the expanding tumor would eventually have compressed the recurrent laryngeal nerve. In determining a treatment strategy, the risks and benefits must be carefully evaluated to select the best approach for the patient.

## Conclusion

We reported a case of paralysis of the recurrent laryngeal nerve after RFA for locally recurrent lung cancer lesion in the aortic arch area. In RFA of the thoracic region, it is necessary to recognize the potential risk of nerve injury depending on the site of ablation and to determine the optimal treatment strategy.

## Patient consent

Written informed consent for publication of this case was obtained from the patient.
